# A promising antimicrobial bionanocomposite based poly(3-hydroxybutyrate-*co*-3-hydroxyvalerate) reinforced silver doped zinc oxide nanoparticles

**DOI:** 10.1038/s41598-022-17470-y

**Published:** 2022-08-22

**Authors:** Mohammad I. Ibrahim, Diya Alsafadi, Khalid A. Alamry, Mohammad Oves, Abeer M. Alosaimi, Mahmoud A. Hussein

**Affiliations:** 1 Department of Chemistry, Faculty of Science, King Abdulaziz University, Jeddah, 21589 Saudi Arabia; 2 Biocatalysis and Biosynthesis Research Unit, Foundational Science Research Division, Royal Scientific Society, Amman, 11941 Jordan; 3 Centre of Excellence in Environmental Studies, King Abdulaziz University, Jeddah, 21589 Saudi Arabia; 4 Department of Biological Science, Faculty of Science, King Abdulaziz University, Jeddah, 21589 Saudi Arabia; 5 Department of Chemistry, Faculty of Science, Taif University, P.O. Box 11099, Taif, 21944 Saudi Arabia; 6 Chemistry Department, Faculty of Science, Assiut University, Assiut, 71516 Egypt

**Keywords:** Materials chemistry, Materials science, Biomaterials

## Abstract

A bionanocomposite based on biosynthesized poly(3-hydroxybutyrate-*co*-3-hydroxyvalerate) (PHBV) and reinforced with silver@zinc oxide (Ag–ZnO) was synthesized in variable loadings of Ag–ZnO using the in-situ casting dissolution technique. The degradable biopolymer PHBV had been biosynthesized from date waste as a renewable carbon source. The fabricated products were investigated as promising antibacterial materials. The Ag–ZnO nanoparticles were also synthesized using the green method in the presence of Gum Arabic. The Ag–ZnO nanoparticles were loaded within the PHBV biopolymer backbone at concentration of 1%, 3%, 5% and 10%, PHBV/Ag–ZnO_(1,3,5,10%)_. The chemical structure, morphology, physical and thermal properties of the PHBV/Ag–ZnO bionanocomposites were assessed via common characterization tools of FTIR, TGA, XRD, SEM and EDX. One step of the degradation process was observed in the range of 200–220 °C for all the obtained materials. The onset degradation temperature of the bionanocomposites have been noticeably increased with increasing the nanofiller loading percentage. In addition, fabricated products were investigated for their interesting antibacterial performance. A detailed biological screening for the obtained products was confirmed against some selected Gram-positive and Gram-negative strains *S. aureus* and *E. coli*, respectively. Overall, the bionanocomposite PHBV/Ag–ZnO_(10%)_ was the most potent against both types of the selected bacteria. The order of bacterial growth inhibition on the surface of the fabricated bionanocomposites was detected as follows: PHBV/Ag–ZnO_(10%)_ > PHBV/Ag–ZnO_(5%)_ > PHBV/Ag–ZnO_(3%)_ > PHBV/Ag–ZnO_(1%)_.

## Introduction

Poly(3‑hydroxybutyrate‑*co*‑3‑hydroxyvalerate) (PHBV) is a biopolyester with favorable properties like improved mechanical properties and lower melting point when compared to Poly(3-hydroxybutyrate) (PHB)^1,2^. Also, it has a wider processing window due to lower glass melting and transition temperatures^3,4^, in addition to less brittleness, lower crystallinity and higher biodegradability as percentage of valerate increases^5,6^. The PHBV is distinguished with its properties like biodegradability, low cytotoxicity, biological origin, thermoplasticity, biocompatibility with many types of cells, piezoelectricity, high degree of crystallinity, solubility in chlorinated solvents, excellent oxygen barrier properties, chemical inactivity, ultraviolet radiation resistivity but still it is a relatively brittle polymer with low impact resistance and poor thermal stability^7–10^. The biocompatibility of PHBV has qualified it to be extensively exploitable for the pharmaceutical and other biomedical applications such as bone scaffolds, drug delivery systems, implant coatings and tissue engineering in general^11^. Regardless of the concerns on PHBV properties and process costs^12^, and in order to overcome these concerns, many synthesized biocomposites—where fillers have enhanced or even totally introduced new features on PHBV- have been investigated^13^. Applications were targeting packaging, biomedical applications, including tissue engineering, drug delivery and others. Most of the published biocomposites had the fillers from bio-origin leading to totally biodegradable composites. PHA applications and their biocomposites were focused in bone scaffolds and tissue engineering^14,15^, therapeutics field^16,17^, their copolymers^18^ and many others^1,19,20^. PHBV biocomposites and their applications were reviewed in literature^13,21,22^. PHBV was very much investigated for packaging applications as well^23–25^. The antimicrobial properties of silver nanoparticles (AgNPs) and zinc oxide (ZnO) have been incorporated into PHBV for food packaging^18–28^. Stabilized silver nanoparticles 0.04 wt% were used in PHBV to produce a nanocomposite with 56% reduction in oxygen permeability when compared to PHBV, and the films showed a prolonged—up to seven months—antimicrobial activity against the pathogens, *Listeria monocytogenes* and *Salmonella enterica*^26^. The use of zinc oxide alone with different morphologies and sizes (micro and nanoparticle sizes) to reinforce PHBV films was reported. Zinc oxide nanoparticles have enhanced the optical and thermal properties of the nanocomposite films in addition to the antimicrobial properties which qualifies it for food packaging^27^. Other examples include the use of zinc oxide with reduced graphene oxide into glycerol-plasticized PHBV to form hybrid nanocomposites that were melt-extruded^29^. PHBV and ZnO nanoparticles were prepared by solvent casting to form composite films where the optimum percentage of ZnO was reported to be 4 wt%. This reported optimum ratio showed the best barrier properties, the maximum Young’s and storage moduli and tensile strength in addition to the highest crystallinity. The antibacterial properties were also confirmed^30^. To enhance mechanical and antimicrobial properties, the extract from the leaves of *Thymus vulgaris* was used for the synthesis of zinc oxide–silver nanocomposites which were incorporated into PHBV-chitosan for the purpose of achieving lowest immigration rate and hence to improve the shelf life of poultry items. Good antimicrobial activity has been reported that qualifies them for food packaging of poultry items^31^. Ternary composites with nanohybrids of silver and cellulose nanocrystals with PHBV via solution casting have been investigated, and higher percentages of silver nanoparticles were associated with better overall migration, mechanical, thermal stability, antibacterial and barrier properties, and suggested as promising materials for food packaging^32^. A nanohybrid of zinc oxide and cellulose nanocrystals with PHBV matrix was synthesized by simple solution casting, the fabricated nanocomposites had antibacterial ratios of 95.2–100% for both bacteria *S. aureus* and *E. coli*. They have also degraded with 9–15% after one week. The incorporation of zinc oxide and cellulose nanocrystals has improved barrier properties and hydrophilicity^33^.

Although the aforementioned studies reported the incorporation of AgNPs and ZnO nanoparticles into PHBV matrices, to the best of our knowledge, no information is available on the incorporation of mixture of Ag doped ZnO nanoparticles in PHBV matrices. Therefore, the main goal of this work was to develop and characterize the antibacterial activity and the physicochemical properties of PHBV bionanocomposites and layer films containing both Ag–ZnO nanoparticles. The Ag–ZnO nanoparticles were synthesized using Gum arabic, and also the PHBV had been biosynthesized by the microorganism *Haloferax mediterranei* utilizing date waste as carbon source.

## Materials and methods

### Materials

Poly(3-hydroxybutyrate-*co*-3-hydroxyvalerate) (PHBV) was produced by *Haloferax mediterranei* via biosynthesis utilizing date waste as carbon source and with high 3-hydroxyvalerate (3 HV) content of 18.0 mol%. Date waste had been purchased from local market at Qassim region in Saudi Arabia. The produced PHBV was of high molecular weight (746.0 kDa), narrow polydispersity (PDI = 1.5) and a melting point at 148.1 °C, the details of the biosynthesis were previously reported^34^.

### Biosynthesis of PHBV polyester

Date waste has been purchased from local market at Qassim region in Saudi Arabia. PHBV had been biosynthesized by halophilic archaeon *Haloferax mediterranei* from date waste biomass as feed-stock, the complete details of biosynthesis and characterization have been previously described in a published research^34^. The study complies with local and national guidelines in Saudi Arabia.

### Synthesis of Ag-doped ZnO nanoparticles using Gum Arabic (GA)

To synthesize Ag–ZnO nanoparticles using Gum Arabic as a green procedure, 50 mL of AgNO_3_ (0.001 M) was added to 50 mL of ZnSO_4_.7H_2_O (0.1 M) and the mixture was stirred for 0.5 h. To this mixture, 40 mL of GA solution (1% (w/v)) was added and the stirring was continued for another 0.5 h. The pH of the reaction mixture was adjusted to pH ~ 10 by drop wise addition of 1 M NaOH solution and the mixture was further stirred for 1.5 h at room temperature. The resulting colloidal solution was aged at room temperature for 24 h and was centrifuged. The product thus obtained was washed well with ethanol and distilled water and was dried at 450 °C in an electric oven. The dried product was calcined for 1 h at 400 °C in a muffle furnace.

### Synthesis of PHBV/Ag–ZnO bionanocomposites

Different amounts of Ag–ZnO (0.007 g, 0.021 g, 0.035 g, 0.07 g) nanoparticles were weighed and added separately to glass tubes with 5 ml CHCl_3_ in each, and the suspensions were sonicated for 15 min. In four 50 ml beakers, 0.7 g of PHBV polymer was dissolved in 18 ml CHCl_3_ in each of the four beakers with stirring for 30 min. The nanoparticle suspensions in CHCl_3_ were poured separately into the four 50 ml beakers containing the PHBV polymer dissolved in CHCl_3_ and the resultant four mixtures was stirred for 5 min. Then the formed suspensions were poured into watch glasses and covered with a glass cover and left to dry. The formed bionanocomposites (1%, 3%, 5% and 10%) were then taken and characterized.

### Instrumentation

#### Scanning electron microscope (SEM) analysis

Samples were analyzed by Dual beam scanning electron microscope model Scios 2 from Thermo Fisher Scientific. Samples were fixed on a carbon holder and then inserted into the SEM chamber to image them under vacuum at different electron beam strength settings.

#### X-ray diffraction

The XRD patterns for the composites were recorded using Panalytica X’pert PRO MPD X-Ray Diffractometer with Cu Kα radiation (λ = 0.15418 nm, 45 kV, 40 mA).

#### Thermogravimetric analysis (TGA)

High resolution dynamic Thermogravimetric analysis (TGA) were performed under a continuous Nitrogen flow and recorded on a TA Instruments Hi-Res TGA Q500 thermogravimetric analyzer with a heating rate of 5 °C per minute.

#### Fourier Transform Infrared (FT-IR)

FT-IR spectra (4000–600 cm^−1^) were collected in the solid state on Nicolet 700 FT-IR Spectrometer-Thermo Fisher Scientific.

### Antimicrobial activity study of PHBV/Ag–ZnO bionanocomposites

#### Surface inhibition assay

The fresh bacterial culture was developed one day before the experiment from the stored lab-cultured isolate for the surface inhibition assay on the media plate surface killing. The colony sample was taken from the reserved cultured plate and was inoculated into the 25 ml broth medium, where it was incubated overnight at 35–37 °C. The culture was diluted the next day and grown back to the mid-log phase (4 h incubation) since the most active phase of the log phase was retained and standardized by absorbance reading 0.5 at 600 nm filter and turbidity in culture was maintained according to the McFarland standard with the help of a colorimeter. During cultivation incubation, a stock solution of test compounds (1, 3, 5, 10% Ag–ZnO) was developed in an aqueous solution, and an antibiotic Amoxicillin was used as a positive monitor. Each compound's stock solutions were formulated at a standard concentration of 100 µg/ml. On the media surface, 5 µg/ml of each compound's solution was put on dry-cultured plates. These cultured plates were made from a new culture of the test strain, spread thinly over the plate, and dried for 10 min in the hooded chamber to allow the test strain to settle and the plating liquid to dry out. In addition, these plates were incubated overnight in the incubator without being inverted. Bacterial development around the compounds was found to be disrupted bacterial growth the next day.

#### MIC and MBC determination

The antimicrobial activity of newly synthesized compounds (1% Ag–ZnO; 3% Ag–ZnO; 5% Ag–ZnO; 10% Ag–ZnO) was calculated by the MIC and MBC using the steps below. *S. aureus* and *E. coli* were chosen as the test microorganisms. Both research- strains were grown overnight in Nutrient Broth (NB) and cultured diluted, with cell density up to 1 × 106 CFU/mL. The stock solution was made separately using composite materials with Ag–ZnO nanofiller percentages of 1, 3, 5 and 10%. Dilutions made in a 1:1 ratio for the test compound and culture tubes are often made in separate test tubes. It entails inoculating all test compound dilutions with equal amounts of the bacterial organisms *E. coli* and *S. aureus* and maintaining positive and negative control test tubes separately to ensure sufficient bacterial growth and media sterility for overnight incubation at 35 °C. An aliquot of the positive control test tubes was spread on a solid agar media plate to confirm a baseline microbial concentration of up to 1 × 106 CFU/mL. With or without compounds, both bacterial culture tubes are incubated for 24 h at 35 °C. The turbidity was not obtained at the lowest concentration, designated as the compound's minimum inhibitory concentration (MIC). The test compound concentration of the observed MIC value calculated as MBC increased by a factor of two or more.

## Results and discussion

### Characterization of PHBV/Ag–ZnO bionanocomposites

The pre-synthesized Ag–ZnO nanoparticles with Ag percentage of 1 mol% were added to PHBV pure polymer to form composites with different Ag–ZnO reinforcements (1, 3, 5 and 10%). The final products have been abbreviated as PHBV/Ag–ZnO(_x%_) where *x* is the percentage of the reinforced Ag–ZnO nanofiller. In order to study the synthesized nanoparticles and the new bionanocomposites characteristics, the samples were tested with FTIR, XRD, SEM, EDX and TGA common characterization tools.

**Figure 1:**
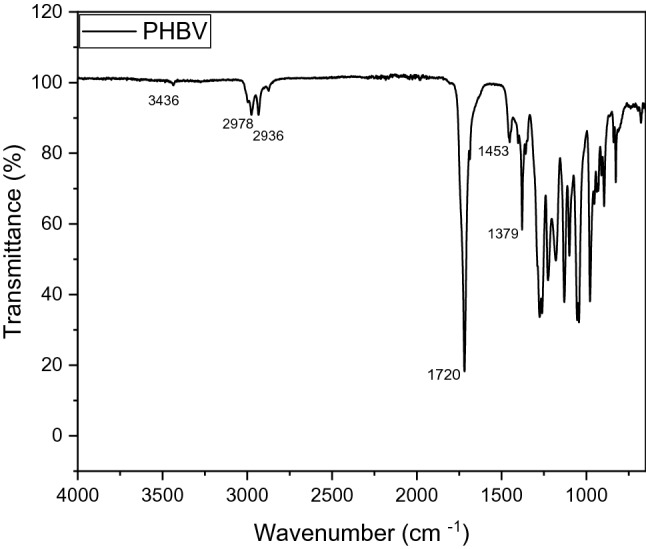
FTIR spectra of neat PHBV.

The FTIR spectrum of the neat PHBV polymer (Fig. 1) shows clearly the characteristic peaks of this polyester; most importantly is the intense peak at 1720 cm^-1^ that corresponds to the ester carbonyl C=O stretching frequency which also appears as intense peak in all the prepared composites with different percentages^34^.

**Figure 2:**
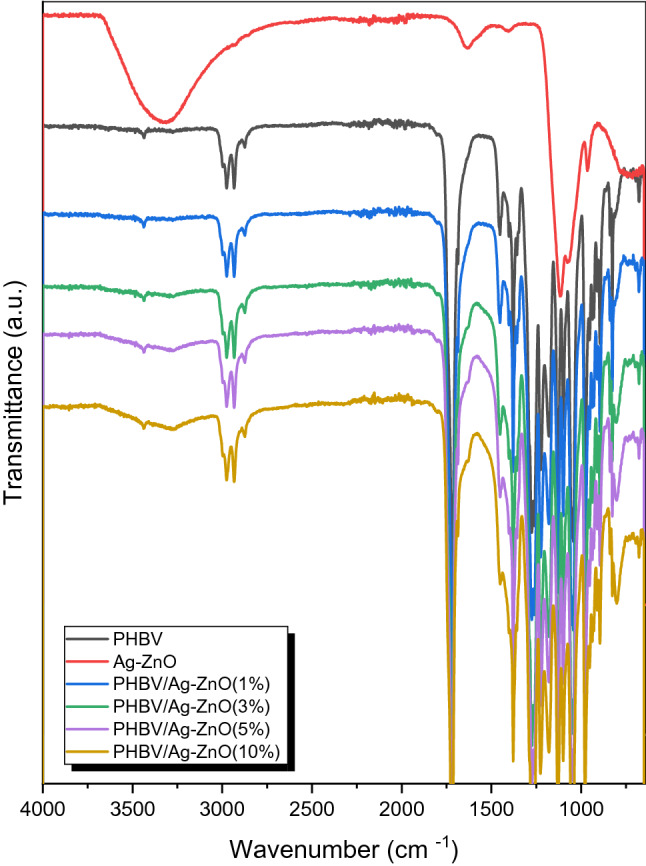
FTIR spectra of pure PHBV Ag–ZnO, and PHBV/Ag–ZnO(_1, 3, 5, 10%_) nanocomposites.

This most intense peak at 1720 cm^-1^ is becoming even more intense in the case of the nanofiller percentages above 3%, with the highest intensity in case of 3% filler composition, this finding is in line with previous findings for a similar research^35^, the carbonyl peak-being a main polar part of the polyester- is expected to significantly contribute to the crystallinity nature of the polymer. Also, it has been reported that the stretching frequency position of the C=O is affected by the strength of interaction between polymer chains, namely the interaction C–H–C=O that results from the interaction between a carbonyl group in one chain and an α-methylene group adjacent to another carbonyl in another chain^35^. Frequencies at 1379 cm^-1^ and 1453 cm^-1^ corresponds to the amorphous part of the polymer^35^. The peaks at 2874 cm^-1^, 2936 cm^-1^and 2978 cm^-1^ corresponds to the different C–H stretching frequencies. Other peaks frequencies assignments are also observed and match with reported^35^. As shown in Fig. 2, the combined spectra of pure PHBV and its composites with different percentages of Ag–ZnO show very similar peaks frequencies at the whole range and this indicates that the PHBV polymer bonds strengths haven’t changed any significantly due to the incorporation of the nanofiller.

**Figure 3:**
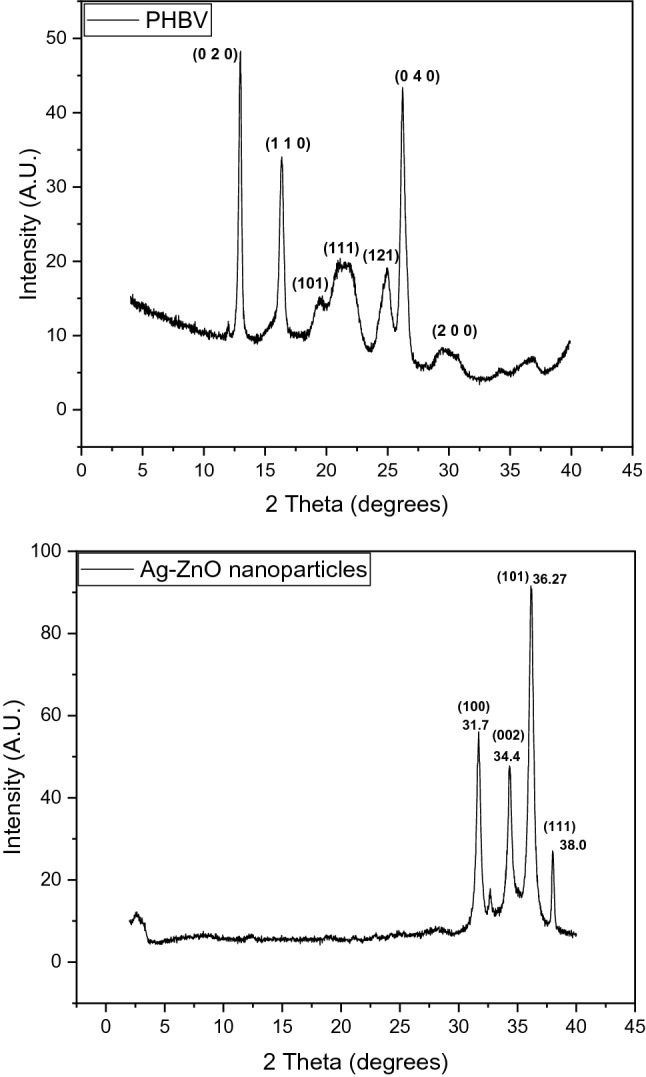
XRD of neat PHBV and Ag–ZnO nanoparticles.

The XRD crystallographic pattern for the pure PHBV copolymer and Ag–ZnO nanofiller is very well matching the expected diffraction patterns for both samples. The pure PHBV pattern shows peaks (020), (110), (101), (111), (121), (040) and (200) which are in accordance with the proposed structure^34^. Furthermore, the crystalline hexagonal quartzite phase and fits with the XRD peaks values as in the Joint Committee on Powder Diffraction Standards (JCPDS) card no JCPDS PDF no 36-1451 as illustrated in Fig. 3.

The XRD peaks in Fig. 3 also shows that, the *2θ* values of 31.7, 34.45 and 36.27 degrees correspond to the ZnO hexagonal quartzite lattice planes (100), (002) and (101), respectively^36^. The incorporation of Ag is confirmed by the diffraction peak at 38.0 degrees which is characteristic to the face-centered-cubic (fcc) phase of metallic Ag of the lattice plane (111) by referring to JCPDS file no. 04-0783.

**Figure 4:**
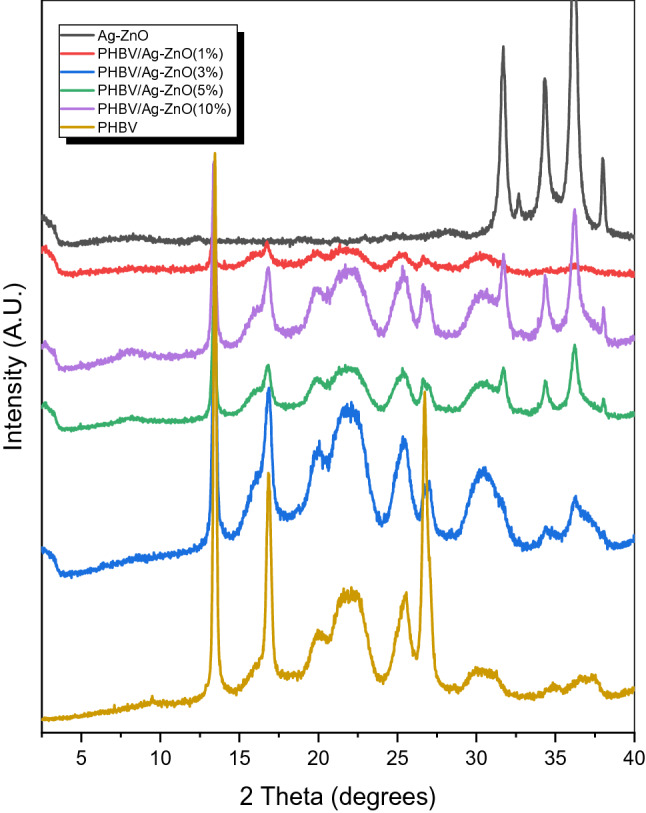
XRD spectra Ag–ZnO, PHBV and PHBV/Ag–ZnO_(1, 3, 5, 10%)_ bionanocomposites.

It has been previously reported that doping ZnO with Ag has an effect on the positions of the (101) and (002) diffractions peaks and their FWHM^36^, the study has reported these peaks of planes (101) and (002) at slightly higher values for samples with higher percentages of Ag doping, and the peaks positions here in this study matches well with the same previous study reported values for the 1% doping of Ag in ZnO. The authors also claimed that the incorporation of Ag within the lattice of ZnO which occurs through Ag^+^ ions substituting Zn^2+^ ions in the ZnO lattice is quite difficult due to the relatively big difference in the ions sizes, (1.22 Å) and (0.74 Å) for Ag^+^ and Zn^2+^, respectively. Another point that is worth-mentioning here is that the existence of the characteristic diffraction peak at 38.0 degrees clearly resembles the existence of crystalline silver clusters in the synthesized nanoparticles, and this is explained by the previous discussion on the size difference between the two ions of Ag^+^ and Zn^+2^. Other XRD measurements were conducted on the neat PHVB polymer and for all the bionanocomposites of PHBV with different percentages of Ag–ZnO nanoparticles. As shown in Fig. 3, the PHBV polymer exhibits the well-known characteristic peaks at 2θ values of 13.7°, 17.1°, 20.4°, 21.5°, 22.7°, 25.5°, 26.9°, 30.6° which correspond to the typical (020), (110), (021), (101), (111), (121), (040), (200) crystal planes for orthorhombic PHBV, respectively^37^. The intense peaks as 13.7° and 17.1° are connected with the crystalline part of the polymer, while the broad peaks at 2θ = 20° to 22° are corresponding to the amorphous phase in the PHBV^35^. Figure 4 shows the combined XRD spectra for neat PHBV in comparison to the Ag–ZnO nanofiller and the fabricated PHBV/Ag–ZnO(_1, 3, 5, 10%_) bionanocomposites.

In order to study the crystallization behavior of the bionanocomposites compared to the neat PHBV, the XRD characteristics peaks of both the neat polymer and its bionanocomposites have been investigated. The investigated diffraction peaks positions of the planes (020), (110) and (040) have shown no significant changes in their positions with a slight increase in peak widths which implies that the formed bionanocomposites are slightly less crystalline than the neat PHBV but are still highly crystalline in nature, and without any noticeable phase changes. On the other hand, a significant decrease in the sharp peaks intensities at 13.7°, 17.1° and 26.9° suggests a considerable decrease in the crystallinity of the bionanocomposites compared to the neat PHBV. Also, as expected, the peaks at 31.7°, 34.45°, 36.27° and 38.0° corresponding to the nanofiller are increasing in intensity with the increased filler percentage. Another important observation is the formation of a new broad and low intensity peak at around 15.9°, indicating the formation of a new crystalline phase^36^.

**Figure 5:**
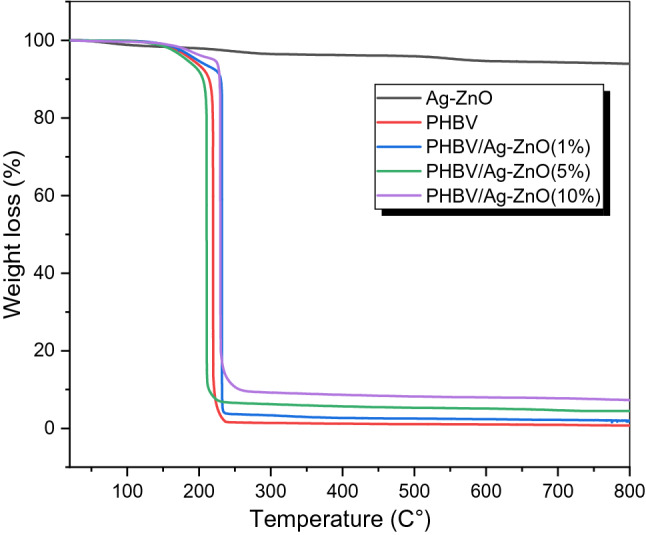
TGA curves for pure PHBV, Ag–ZnO, and PHBV/Ag–ZnO_(1, 5, 10%)_ bionanocomposites.

Additionally, samples of neat PHBV, Ag–ZnO nanoparticles and their bionanocomposites PHBV/Ag–ZnO_(1, 5, 10%)_ have been all analyzed by thermogravimetric analyzer in the temperature region of RT to 800 °C. As can be seen in Fig. 5, it was depicted from the analyses that peak degradation temperatures have been shifted towards higher values for the composites of 1% and 10%, indicating a better thermal stability for the polymer by introducing these percentages of the filler. One step degradation step has been observed in the range of 200–220 °C for all the tested products. Also, the onset degradation temperature has been noticeably increased in case of the composite with 10% filler. Noticeable increase in the thermal stability has been investigated in comparable to the pure copolymer. This general increases in the thermal stability of the pure biosynthesized copolymer can be attributed to the incorporation of the metal oxide Ag–ZnO between the chains with possible bonding between metal part from the filler and the carbonyl groups in the neat copolymer.

**Figure 6:**
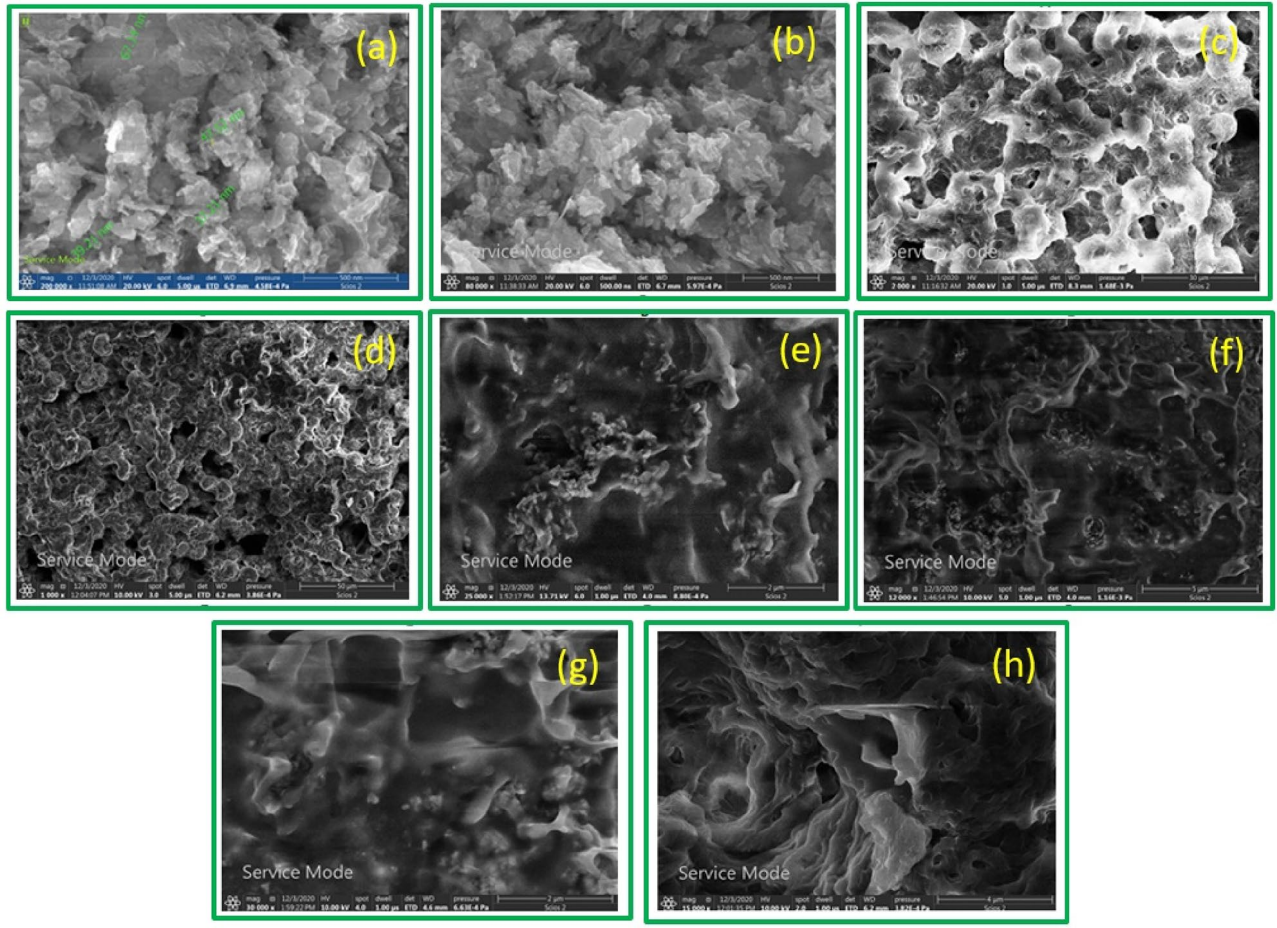
SEM micrographs of Ag–ZnO nanoparticles at magnifications of (**a**) x = 200,000, (**b**) x = 80,000; PHBV/Ag–ZnO_(1%)_ at magnifications of (**c**) x = 2000, (**d**) x = 1000; PHBV/Ag–ZnO_(5%)_ at magnifications of (**e**) x = 25,000, (**f**) x = 12,000; PHBV/Ag–ZnO_(10%)_, at magnifications of (**g**) x = 30,000, (**h**) x = 15,000.

**Figure 7:**
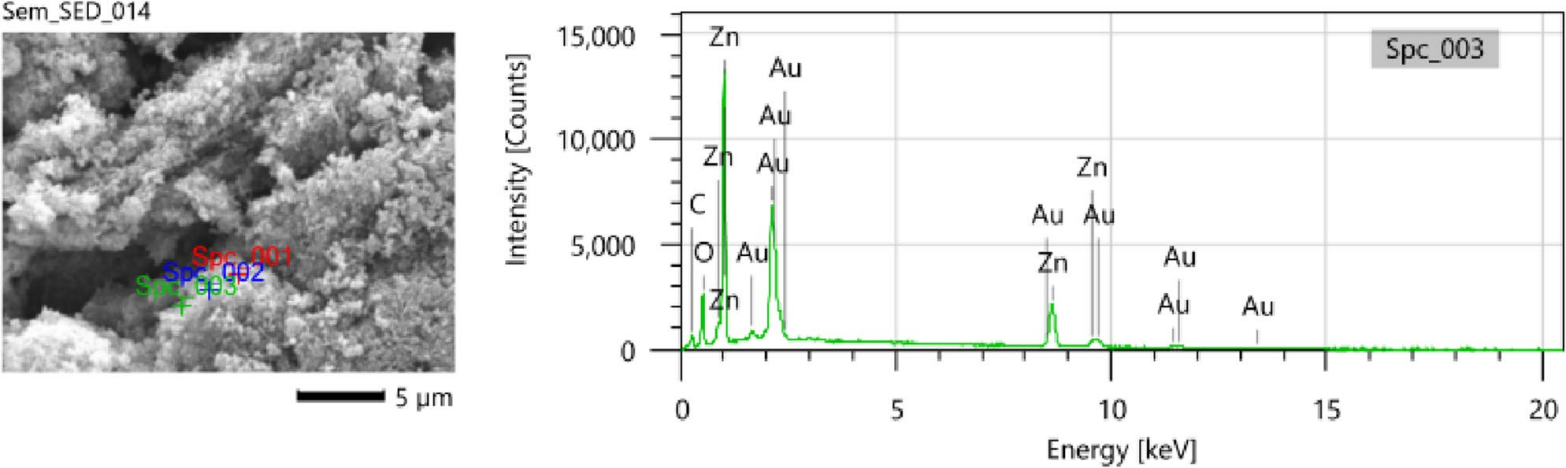
EDX analysis of PHBV/Ag–ZnO_(5%)_ bionanocomposite. The Au peaks are due to the gold coating used for SEM sample preparation. Ag peaks are not visible, as Ag doping was constant at 1 mol% which is below the detection limit.

The SEM images of the Ag–ZnO nanoparticles and PHBV/Ag–ZnO_(1,5,10%)_ bionanocomposites, are illustrated in Fig. 6. The typical Ag–ZnO nanoparticles images are given in Fig. 6a,b. Micro forest structures of flower like porous ZnO were observed. Whereas, the different compositions of Ag–ZnO reinforced to pure PHBV copolymer chains are displayed in Fig. 6c–h. These images confirm the homogenous and uniform distribution of Ag–ZnO nanoparticles within the copolymer matrix. In addition, the Ag–ZnO nanoparticles size in the bionanocomposites ranging from 40 to 70 nm in a rod-like shapes that are unevenly distributed within the PHBV cavities. Also, the existence of Zn has been confirmed by EDX analysis of specific spots during SEM imaging of PHBV/Ag–ZnO_(5%)_ bionanocomposite as a selected example as illustrated in Fig. 7. Ag peaks are not visible as its doping percentage is below the detection limit. The Au peaks are due to the gold coating used for SEM sample preparation.

## Antimicrobial activity of PHBV/Ag–ZnO bionanocomposites

### Bacterial surface inhibition

**Figure 8:**
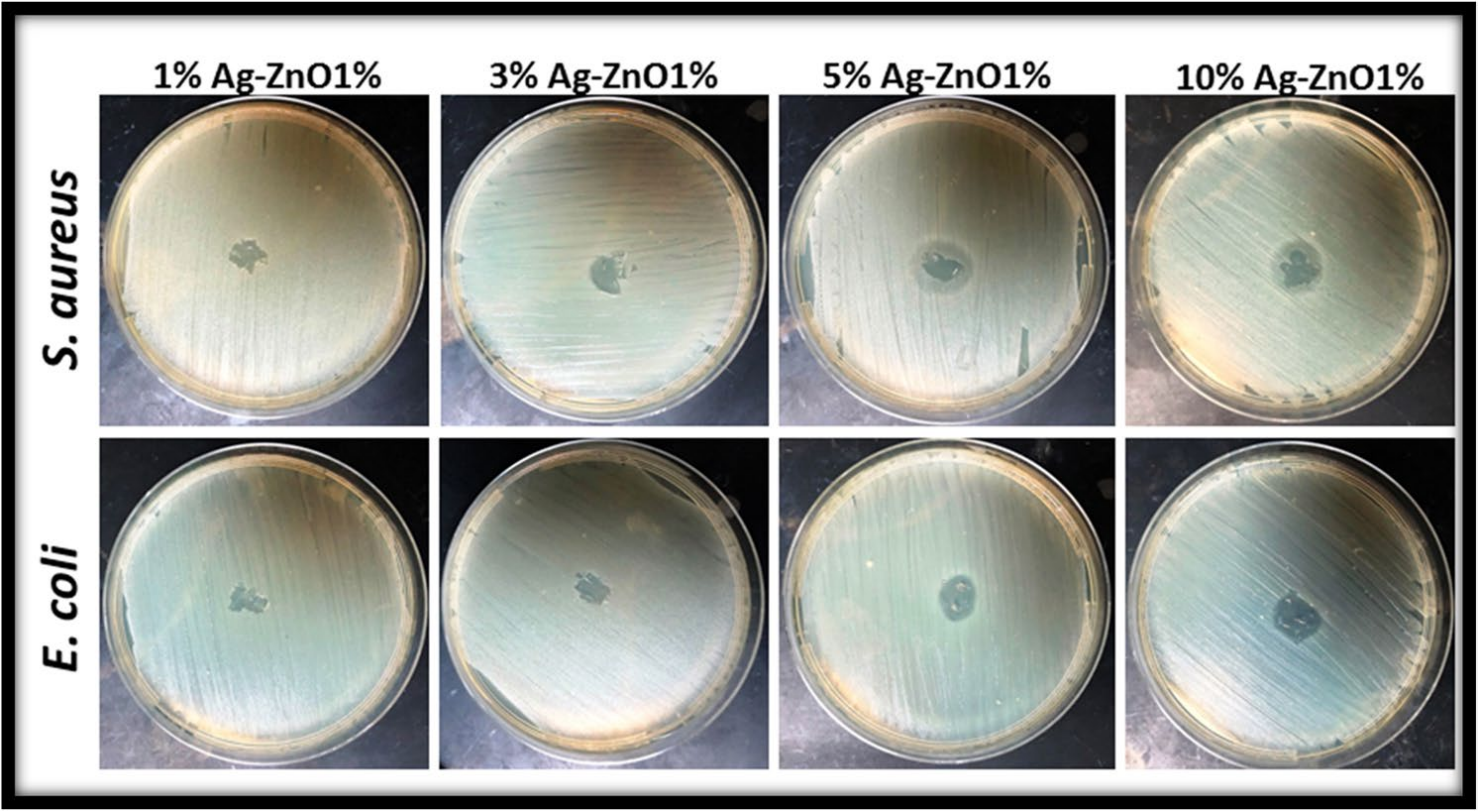
The surface bacterial growth inhibition by the applied PHBV/Ag–ZnO_(1,3,5,10%)_ bionanocomposites against the *S. aureus* and *E. coli* as a bacterial test strain.

The surface inhibition assay is a critical parameter for the determination of bacterial resistance to compounds added to the media layer. They are monitored to interact with increasing microbes and visible patterns are observed. The Kirby and Bauer disk diffusion assay has been updated for this process. Without automatic tests, it is a feasible alternative to the broth dilution method of substance testing. Without using a filter paper disk impregnated compound, a known concentration of test compound (5 µg) was directly added to the surface of the cultured plate, where it absorbed the water from the agar and diffused the surrounding compounds. During the incubation period, the compound interacted with the culture bacteria. When an antimicrobial compound is present at the critical point of diffusion, bacteria's growth at critical biomass is blocked, and a clear zone forms around the applied bionanocomposite. As expected, and shown in Fig. 8, the bionanocomposite with 10 wt% Ag–ZnO (PHBV/Ag–ZnO_(10%)_) created a maximal zone of 13 and 14 mm inhibition against gram-positive *S. aureus* and Gram-negative *E. coli* bacterial stains, respectively. The order of antimicrobial action against bacterial strains in the sequence of composites according to the increasing percentage of the added filler of Ag–ZnO against both bacterial test strain *S. aureus* and *E. coli*, the order of bacterial growth inhibition on the surface PHBV/Ag–ZnO_(10%)_ > PHBV/Ag–ZnO_(5%)_ > PHBV/Ag–ZnO_(3%)_ > PHBV/Ag–ZnO_(1%)_. Overall, the bionanocomposite with PHBV/Ag–ZnO_(10%)_ was the most potent against both types of the bacterial strains.

## MIC and MBC determination

The minimum inhibitory concentration was calculated using the broth dilution process, which involved sub-culturing compound-treated bacteria onto agar plates using the spread plate technique without changing the assay agent in the plate. However, MIC is the lowest antibacterial compound concentration that decreases the viability of the original bacterial inoculum by at least 99.9%. The MBC is the minimum bactericidal concentration which is the lowest concentration of test compound that destroys a bacterium in a fixed-dose or comparatively long period, such as 24 h, under optimal conditions. Overall, the MBC is a supplement to or expansion of the MIC test; moreover, the MIC test determines the lowest level/concentration/dose of test compound that significantly inhibits bacterial development. The tested composite is considered bactericidal if its MBC value does not surpass four times the MIC value. The MBC test can be used to assess the potency of a drug and to rule out issues during formulation, such as the active ingredient being bound to other compounds and thus unable to kill bacteria in the media. Gram-positive bacteria (*S. aureus*) were found to be more susceptible than Gram-negative bacteria (*Escherichia coli*) in this research and this was in line with previously reported research which showed that *E. coli* is more resistant to ZnO than *S. aureus*^38^*.* The bionanocomposite of PHBV/Ag–ZnO_(10%)_ was the most effective antibacterial composite against all types of bacteria when synthesized composites were used as antimicrobial agents. The bionanocomposite with PHBV/Ag–ZnO_(10%)_ had MIC and MBC levels of 15 and 30 µg/ml against *E. coli* and 10 and 20 g/ml against *S. aureus*, respectively. The bionanocomposites x% Ag–ZnO antimicrobial property in MIC and MBC against *S. Aureus* and *E. coli* are depicted in Table 1. As MIC and MBC findings were compared, PHBV/Ag–ZnO_(1%)_ was the least toxic against both bacterial strains, and hence the conclusion that the antibacterial potential increases with increased percentage of the Ag–ZnO nanofiller concentration in the bionanocomposite.

**Table 1:** Antimicrobial properties of synthesized PHBV/Ag–ZnO_(1, 3, 5, 10%)_ bionanocomposites.

Microorganisms/composite	Gram-negative		Gram-positive	
	*E. coli*		*S. aureus*	
	MIC	MBC	MIC	MBC
PHBV/Ag–ZnO_(1%)_	50	100	40	80
PHBV/Ag–ZnO_(3%)_	40	80	30	60
PHBV/Ag–ZnO_(5%)_	25	50	20	40
PHBV/Ag–ZnO_(10%)_	15	30	10	20

## Conclusion

Pre-synthesized Ag@ZnO nanoparticles with Ag percentage of 1 mol% had been successfully composited with biosynthesized PHBV copolymer to form novel bionanocomposites with different percentages of Ag–ZnO reinforcement (1, 3, 5 and 10%) and were used for the fabrication process with given abbreviations of PHBV/Ag–ZnO_(1,3,5,10%)_. The synthesized bionanocomposites had been characterized by FTIR, XRD, SEM and EDX which confirmed the chemical structures for the end products. The thermal behavior had been studied by TGA thermal analysis. All the products gave the same degradation profile. A fast one step of degradation thermogram had been seen for the fabricated PHBV/Ag–ZnO_(1,3,5,10%)_. The antibacterial performance of the casted bionanocomposites was confirmed by the study against selected Gram positive and Gram negative strains (*S. aureus* and *E. coli*), respectively. The order of antimicrobial action against bacterial strains was found in the sequence of composites according to the increasing percentage of the added filler of Ag–ZnO against both bacterial test strains *S. aureus* and *E. coli*. The maximal observed inhibition zone against gram-positive *S. aureus* and Gram-negative *E. coli* bacterial strains was associated with PHBV/Ag–ZnO_(10%)_ which gave 13 mm and 14 mm inhibition diameter, respectively. The synthesized bionanocomposites are considered of great interest in the development and design of biodegradable active packaging materials for food industry.

## Data Availability

All data generated or analyzed during this study are included in this published article.
