# Author Correction: A promising antimicrobial bionanocomposite based poly(3-hydroxybutyrate-*co*-3-hydroxyvalerate) reinforced silver doped zinc oxide nanoparticles

**DOI:** 10.1038/s41598-023-28886-5

**Published:** 2023-01-31

**Authors:** Mohammad I. Ibrahim, Diya Alsafadi, Khalid A. Alamry, Mohammad Oves, Abeer M. Alosaimi, Mahmoud A. Hussein

**Affiliations:** 1 Department of Chemistry, Faculty of Science, King Abdulaziz University, Jeddah, 21589 Saudi Arabia; 2 Biocatalysis and Biosynthesis Research Unit, Foundational Science Research Division, Royal Scientific Society, Amman, 11941 Jordan; 3 Centre of Excellence in Environmental Studies, King Abdulaziz University, Jeddah, 21589 Saudi Arabia; 4 Department of Biological Science, Faculty of Science, King Abdulaziz University, Jeddah, 21589 Saudi Arabia; 5 Department of Chemistry, Faculty of Science, Taif University, P.O. Box 11099, Taif, 21944 Saudi Arabia; 6 Chemistry Department, Faculty of Science, Assiut University, Assiut, 71516 Egypt

Correction to: *Scientific Reports* 10.1038/s41598-022-17470-y, published online 22 August 2022

The original version of this Article contained errors.

In the Results and discussion,

“Also, the existence of Ag and Zn has been additionally confirmed by EDX analysis of specific spots during SEM imaging of PHBV/Ag–ZnO_(5%)_ bionanocomposite as a selected example as illustrated in Fig. 7.”

now reads:

“Also, the existence of Zn has been confirmed by EDX analysis of specific spots during SEM imaging of PHBV/Ag–ZnO_(5%)_ bionanocomposite as a selected example as illustrated in Fig. 7. Ag peaks are not visible as its doping percentage is below the detection limit. The Au peaks are due to the gold coating used for SEM sample preparation.”

In addition, the caption of Figure 7 in the original version of this Article was incomplete.

The caption of Figure 7,

“EDX analysis of PHBV/Ag–ZnO_(5%)_ bionanocomposite.”

now reads:

“EDX analysis of PHBV/Ag–ZnO_(5%)_ bionanocomposite. The Au peaks are due to the gold coating used for SEM sample preparation. Ag peaks are not visible, as Ag doping was constant at 1 mol% which is below the detection limit.”

The Article has been corrected.

